# Stereotactic Radiosurgery for Trigeminal Neuralgia Caused by Vertebrobasilar Compression: A Report of Four Cases

**DOI:** 10.7759/cureus.52880

**Published:** 2024-01-24

**Authors:** Yoshimasa Mori, Yasuhiro Matsushita, Kazuyuki Koyama, Atsuo Masago

**Affiliations:** 1 Radiation Oncology, Center for Advanced Image-Guided Radiation Therapy, Shin-Yurigaoka General Hospital, Kawasaki, JPN; 2 Neurosurgery, Gamma Knife Center, Ookuma Hospital, Nagoya, JPN; 3 Neurosurgery, Aoyama General Hospital, Toyokawa, JPN; 4 Radiation Oncology, Gamma Knife Center, Ookuma Hospital, Nagoya, JPN

**Keywords:** trigeminal nerve, vertebral artery, basilar artery, stereotactic radiosurgery, trigeminal neuralgia

## Abstract

Background: Microvascular decompression (MVD) of the trigeminal nerve is an effective procedure for treating patients with trigeminal neuralgia (TGN). However, vertebrobasilar decompression involves technical difficulties and demonstrates a higher risk of minor trigeminal hypesthesia/hypalgesia, transient diplopia, and hearing loss. Stereotactic radiosurgery (SRS) has been an effective alternative treatment for TGN. Few studies reported the treatment results of SRS for TGN caused by vertebrobasilar compression. This report presents the treatment results of SRS using gamma knife (GK) in four TGN cases.

Materials and methods: GK-SRS was performed for TGN due to vertebrobasilar compression in four patients, including two males and two females, aged 67-90 years. The maximum dose of 80 Gy was delivered at the retrogasserian portion (RGP) of the ipsilateral trigeminal nerve root.

Results: All four cases with TGN achieved relief in four to 10 months after GK-SRS. However, TGN recurred 41 months after GK-SRS in one of the four cases. A second GK-SRS at the root entry zone (REZ) at a maximum dose of 70 Gy relieved pain again 10 days later. TGN in another case among the four partially recurred in three years but did not deteriorate until the patient died from old age 62 months after GK-SRS. The other three cases, including the one with repeat GK-SRS, were alive with complete TGN remission at the end of follow-up of 20-52 months. GK-SRS-related adverse effects were not observed in any case.

Conclusions: GK-SRS was a safe and effective treatment in all four TGN cases due to vertebral artery (VA)-basilar artery (BA) compression, although a second treatment session was added again for pain recurrence in one of the four cases.

## Introduction

Neurovascular compression at the cisternal segment of the trigeminal nerve is considered the most prominent cause of trigeminal neuralgia (TGN). Branches of the distal vertebrobasilar trunk or regional veins caused vascular compression in most cases. A large TGN series reported symptomatic TGN compression caused by either the vertebral artery (VA) or the basilar artery (BA) in only 0%-7.7% of cases [1]. Microvascular decompression (MVD) of the trigeminal nerve is an effective operation for treating TGN, with long-term cure rates reported in 69%-96% of cases [1]. However, vertebrobasilar decompression involves technical difficulties and demonstrates a higher risk of minor trigeminal hypesthesia/hypalgesia, transient diplopia, and hearing loss than standard MVD of the trigeminal nerve [1,2]. Stereotactic radiosurgery (SRS), using Gamma Knife (GK), CyberKnife, or other linear accelerator modalities, has been an effective alternative treatment for TGN [3,4]. However, only a few studies reported on the treatment results of SRS for TGN caused by vertebrobasilar compression [5,6]. This study reports treatment results of GK-SRS in four such cases.

## Case presentation

Four cases (two females and two males aged 67-90 years old) were treated with GK-SRS (Table 1).

**Table 1:** Case characteristics of four GK-SRS cases for TGN *CBZ caused a rash and pregabalin was ineffective **Retreatment MVD, microvascular decompression; CBZ, carbamazepine; GK-SRS, Gamma Knife stereotactic radiosurgery; VA, vertebral artery; BA, basilar artery; RGP, retrogasserian portion; REZ, root entry zone; TGN, trigeminal neuralgia

Case	Age/sex	Neuralgia	Medication	Prior MVD	Offending artery	GK-SRS	
						Target	Max. dose
1	90/F	Rt.V1-2	CBZ	no	VA-BA	RGP	80 Gy
2	69/F	Rt.V1-2	CBZ	yes	VA	RGP	80 Gy
3	68/M	Lt.V1-2	none*	no	VA	RGP	80 Gy
						REZ**	70 Gy
4	67/M	Rt.V2-3	CBZ, pregabalin	no	VA	RGP	80 Gy

**Figure 1:**
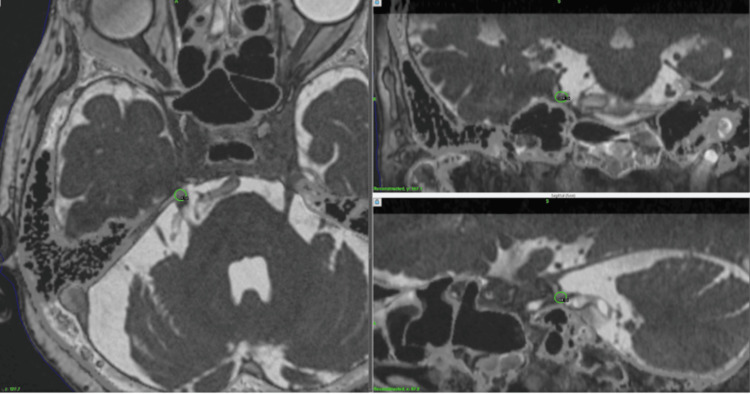
Dose planning for Case 1 MRI (left, axial image; upper right, coronal; lower right; sagittal) on GammaPlan (Elekta, Tokyo) workstation. Radiosurgery shot placement was indicated in green circles, in each image. MRI, magnetic resonance images

**Figure 2:**
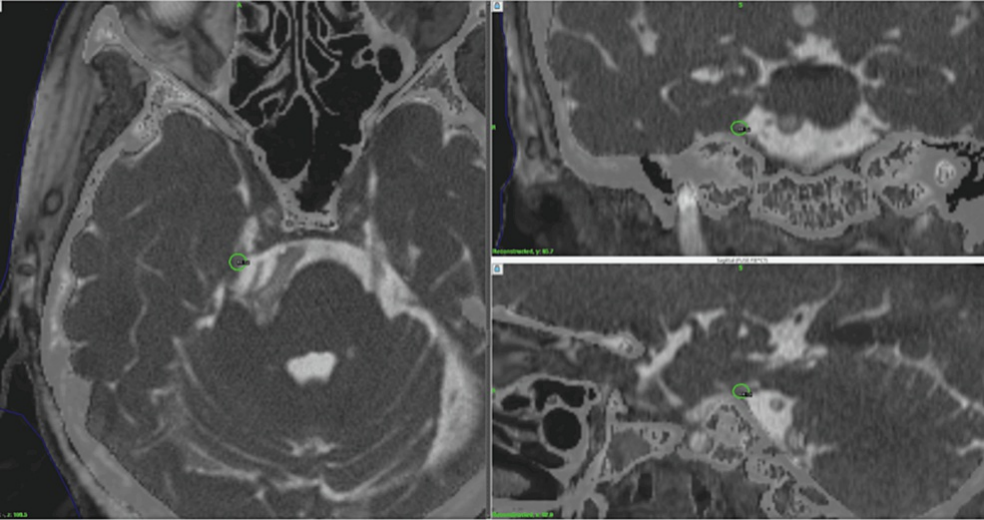
Dose planning for Case 2

**Figure 3:**
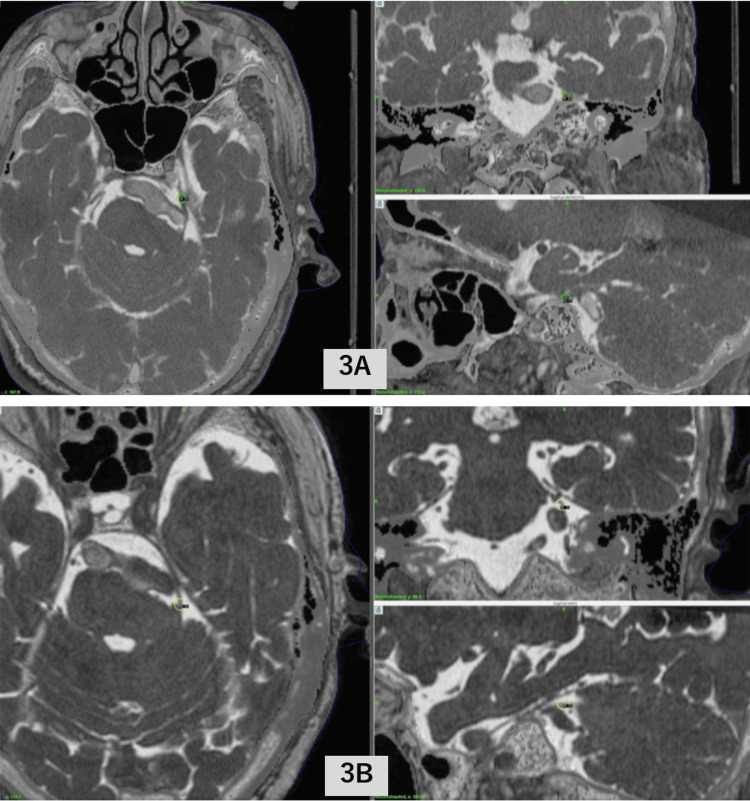
Dose planning for Case 3 3A: first GK-SRS; 3B: second GK-SRS GK-SRS, Gamma Knife stereotactic radiosurgery

**Figure 4:**
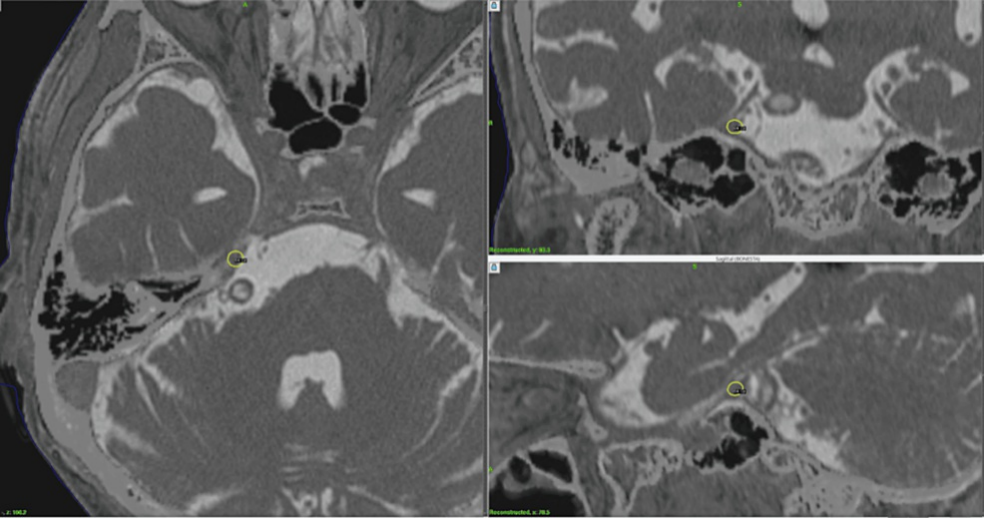
Dose planning for Case 4

Medication had been previously attempted in all four cases but was not fully effective. MVD had been previously performed but failed to improve TGN in one case (Case 2). After MVD, she suffered from an ipsilateral mild facial spasm and thus botulinum toxin therapy followed. Another case (Case 4) experienced a mild facial spasm. A maximum dose of 80 Gy was delivered at the retrogasserian portion (RGP) for GK-SRS in all four cases (Figures 1, 2, 3, 4).

**Figure 5:**
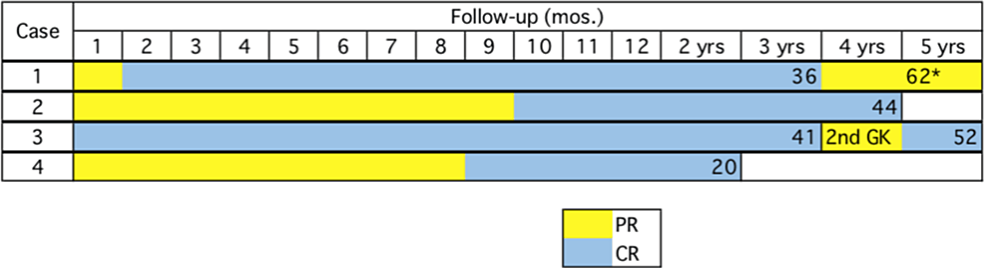
Clinical treatment results of GK-SRS in four cases *The patient died from old age 62 months after GK. GK-SRS, Gamma Knife stereotactic radiosurgery; PR, partial response (partial pain relief); CR, complete response (complete pain disappearance)

All four cases achieved complete TGN remission at 2, 10, 1, and 9 months after GK-SRS, respectively (Figure 5).

A second GK-SRS session was performed for pain recurrence (41 months after the first GK-SRS) three years after the first GK-SRS in one of the four cases (Case 3). The second GK-SRS (Figure 3B) was again fully effective, which helped in complete pain disappearance approximately 10 days after the second GK-SRS. The root entry zone (REZ) was selected as the target for the second GK-SRS with a maximum dose of 70 Gy (Case 3). One of four patients developed partial TGN recurrence three years after GK-SRS but did not deteriorate until death from old age at 62 months after GK-SRS. The other three cases were alive with complete TGN remission at the end of follow-up periods of 44, 52, and 20 months, respectively. None of the cases demonstrated GK-SRS-related adverse effects. 

## Discussion

GK-SRS is an effective treatment option for TGN. A controversy arose over the two different target positionings, REZ and RGP [3,7]. RGP is a little more apart from the brainstem than REZ, and adverse effects on the brainstem would be spared, some reports said [3]. We selected RGP as the target in our four cases. Additionally, REZ was selected for pain recurrence in one (Case 3) of the four cases because changing the target was thought to be better for reducing the probability of adverse effects [8,9]. All five sessions were effective, permanently or temporally.

Only a few studies reported the effectiveness of SRS for TGN caused by vertebrobasilar compression [5,6] (Table 2).

**Table 2:** Reported series of treatment results of GK-SRS for TGN due to vertebrobasilar compression *Facial sensory loss (2/20 cases) (one, permanent; one transient) **Facial sensory loss (3/30 cases) FU, follow-up; TGN, trigeminal neuralgia; GK-SRS=Gamma Knife stereotactic radiosurgery

Series	No. of cases	Dose (Gy)	FU (mos.)	Initial pain relief	Recurrence rate	Adverse effects	Pain-free rate
Park K-J et al., 2012 [5]	20	Median 80 (75-85)	Median 29 (8-123)	75%	60%	10%*	53% at 1 yr, 38% at 2 yrs, 10% at 5 yrs
Tuleasca C et al., 2014 [6]	29	Median 90 (80-90)	Median 46 (13-158)	100%	24%	13%**	76% at 1-5 yrs
Ours, 2023	4	80	Median 48 (20-62)	100%	50%	0	100% at 1 yr (4/4), 100% at 2 yrs (3/3), 0% at 5 yrs (0/1)

Park et al. and Tuleasca et al. revealed initial pain-free rates of 75% and 100% in 20 and 29 patients, respectively. The recurrence rates were 60% and 24%, respectively [5,6]. They reported that the adverse effects were only trigeminal dysfunction. Park et al. revealed 10% (2/20) of transient or permanent facial sensory loss [5]. Tuleasca et al. demonstrated 13% (3/29) of facial sensory loss [6]. Our four cases demonstrated no adverse effects. 

Moreover, only a few studies reported the effectiveness of MVD for TGN caused by vertebrobasilar compression [1,2,10] (Table 3).

**Table 3:** Reported series of treatment results of MVD surgery for TGN due to vertebrobasilar compression *Facial hypesthesia ** 41%, minor trigeminal hypesthesia hypanalgesia; 23%, transient diplopia; 13%, transient hearing loss *** 12.5%, hearing loss; 12.5%, temporary facial paresis and hearing loss FU, follow-up; TGN, trigeminal neuralgia; MVD, microvascular decompression

Series	No. of cases	Surgery	FU (mos.)	Initial pain relief	Recurrence rate	Adverse effects	Pain relief rate
Miyazaki S et al., 1987 [2]	45	MVD	Mean 19 (6-50)	95.60%	4.40%	11%* (51% incl. transient)	ND
Linskey ME et al., 1994 [1]	31	MVD	Mean 60 (1-180)	100%	24%	41%**	96% at 1 yr (92% at 3 yrs), 86% at 5 yrs
Vanaclocha V et al., 2016 [10]	8	MVD	56.5 (14-117)	100%	0%	12.5%***	(100%)

Miyazaki et al., Linskey et al., and Vanaclocha et al. revealed the results of MVD in 45, 31, and 8 cases, respectively [1,2,10]. Initial pain relief was 95.60-100%, though the recurrence rate was up to 24%. However, adverse effects developed in 11%-41% of patients, including not only facial sensory dysfunction but also diplopia and hearing loss. MVD for vertebrobasilar compression requires technical difficulties.

Tuleasca et al. reported in a review that the range intervals for time to pain relief after TGN SRS varied from zero to 16 months (480 days) [3]. In our cases, the latency period also varied from one to 10 months. Tuleasca et al. also reported that later pain relief maintenance following TGN SRS has been positively associated with new facial numbness and no past surgery and negatively associated with a better initial response [3]. In one (Case 3) of our four cases, pain recurred. Case 3 had developed a better initial response.

## Conclusions

GK-SRS was a safe and effective treatment in all four cases of TGN due to VA-BA compression, although a second treatment session was added again for pain recurrence in one of the four cases. Microsurgery of vascular decompression caused by VA or BA includes technical difficulties and carries a higher risk of not only the trigeminal nerve but also neighboring cranial nerves. Therefore, GK-SRS would be an alternative treatment for TGN due to VA-BA compression, though it needs a certain latency period until pain relief.
